# Changes in plasma CGRP levels and clinical outcomes following acupuncture in episodic migraine: a prospective pilot study (the AcuCGRP study)

**DOI:** 10.3389/fneur.2026.1946490

**Published:** 2026-10-16

**Authors:** Valentina Favoni, Lorenzo Giusti, Fiorina Bartiromo, Alessandra Poini, Calogero Calabrò, Elena Merli, Barbara Polischi, Flavia Baccari, Corrado Zenesini, Davide Mascarella, Giulia Pierangeli, Carlo Maria Giovanardi, Sabina Cevoli

**Affiliations:** 1 IRCCS Istituto delle Scienze Neurologiche di Bologna, Bologna, Italy; 2 Neurologia ASL Teramo, Teramo, Italy; 3 Association of Medical Acupuncturists of Bologna, Research and Healthcare Institute, Bologna, Italy; 4 Azienda USL di Bologna, Bologna, Italy; 5 Department of Biomedical and Neuromotor Sciences (DiBiNeM), University of Bologna, Bologna, Italy

**Keywords:** acupuncture, biomarker, CGRP, migraine, migraine prophylaxis

## Abstract

**Background:**

Manual acupuncture is an established non-pharmacological treatment for migraine, with efficacy comparable to conventional prophylactic therapies. Calcitonin gene-related peptide (CGRP) plays a central role in migraine pathophysiology and has been proposed as a biomarker for treatment response. This study aimed to investigate changes in plasma CGRP levels in patients with episodic migraine undergoing a course of manual acupuncture.

**Methods:**

Fifty-two patients with episodic migraine received 10 sessions of standardized manual acupuncture. Clinical assessments and plasma samples were collected at baseline and one week after treatment completion. The primary outcome was the change in plasma CGRP levels. Secondary outcomes included changes in migraine frequency, pain intensity, acute medication intake, and HIT-6.

**Results:**

No statistically significant change in plasma CGRP levels was observed (baseline: 60.9 pg/ml; after treatment: 62.4 pg/ml; *p* = 0.841). Significant clinical improvements were observed, in migraine frequency (median decrease of 1.5 days/month, *p* < 0.001), pain intensity (1-point decrease on a 10-point scale, p < 0.001), acute medication use (2 units/month, *p* < 0.001), and HIT-6 score (−6 points; *p* < 0.001).

**Conclusions:**

This is the first study to investigate changes in plasma CGRP levels following acupuncture in patients with episodic migraine. While CGRP levels remained unchanged, patients experienced reductions in headache frequency, intensity, medication use, and disability. Although these improvements may be attributable, at least in part, to acupuncture, the single-arm design precludes causal inference. Peripheral plasma CGRP may not be a straightforward biomarker of clinical changes in this setting. Controlled studies are warranted.

## Introduction

Migraine affects more than one billion people worldwide and is one of the leading causes of years lived with disability, especially in individuals under 50 years of age (1). The recurrent nature of migraine attacks, combined with their associated symptoms such as nausea, photophobia, and phonophobia, imposes a substantial burden on patients and healthcare systems (2). The treatment of migraine is based on the use of acute and preventive therapies.

Preventive treatments are recommended for patients with frequent or disabling migraine. However, pharmacological prophylaxis may often be limited by adverse effects and poor adherence (3). In this context, manual acupuncture has emerged as a non-pharmacological preventive option with promising results. Several randomized controlled trials and meta-analyses have shown that acupuncture is at least as effective as conventional oral drug prophylaxis in reducing migraine frequency and severity, with a more favorable safety profile (4–10).

At the neurobiological level, calcitonin gene-related peptide (CGRP) plays a central role in migraine pathophysiology. CGRP is a key neurotransmitter in the trigeminovascular system, and its plasma levels are elevated during migraine attacks. The development of CGRP-targeting therapies, such as monoclonal antibodies and gepants, has further highlighted its importance in migraine. However, although the role of CGRP as a therapeutic target is well established, the clinical significance of circulating peripheral CGRP concentrations as a biomarker of migraine activity or treatment response remains uncertain (11–14). In particular, a recent large-scale registry, which measured plasma CGRP using a validated high-affinity radioimmunoassay, found no significant adjusted differences in plasma CGRP concentrations among individuals with episodic migraine, chronic migraine, and healthy controls (15). However, whether circulating CGRP changes longitudinally following therapeutic interventions remains unclear, particularly for non-pharmacological treatments, whose biological mechanisms in migraine are incompletely understood.

A recent randomized, sham-controlled trial of acupuncture in patients with migraine without aura reported no significant between-group difference in changes in serum CGRP levels, although methodological details regarding blood sampling and CGRP measurement were not reported (16). Whether acupuncture may influence circulating CGRP concentrations therefore remains incompletely understood. Exploring longitudinal changes in circulating CGRP following acupuncture may contribute to a better understanding of its potential biological mechanisms in migraine.

The present study is a single-center, pilot observational investigation aiming to assess plasma CGRP levels before and after a 10-session course of manual acupuncture in patients with episodic migraine.

## Methods

### Participants and study design

This single-center, prospective, longitudinal pilot study involved the consecutive enrolment of patients diagnosed with episodic migraine who were referred to the tertiary Headache Center of IRCCS Institute of Neurological Sciences of Bologna, Italy, and deemed eligible for acupuncture as a preventive treatment. Recruitment was conducted between March 2021 and May 2024 by neurologists specialized in headache disorders.

*Inclusion criteria were:* (i) Age between 18 and 75 years; (ii) Diagnosis of episodic migraine, with or without aura, according to the third edition of the International Classification of Headache Disorders (ICHD-III) (17); (iii) A stable frequency of migraine attacks, requiring preventive treatment, as recorded in the headache diary over the three months prior to enrolment; (iv) History of fewer than two prior prophylactic treatments; (v) No preventive treatment or stable preventive treatment during the three months prior to enrolment, with no changes expected throughout the study; (vi) No history of manual acupuncture treatment within the six months prior to enrolment.

*Exclusion criteria were:* (i) Pregnancy or breastfeeding; (ii) Failure of more than two preventive treatments; (iii) Contraindications to acupuncture, such as needle phobia or coagulation disorders; (iv) Use of anti-CGRP treatments during the study period or within the five months preceding enrolment.

The acupuncture intervention was reported in accordance with the STRICTA (Standards for Reporting Interventions in Clinical Trials of Acupuncture) guidelines (18).

The core acupuncture prescription consisted of bilateral LR3 (Taichong), GB34 (Yanglingquan), SP6 (Sanyinjiao), LI4 (Hegu), and TE5 (Waiguan), together with GV20 (Baihui). Additional acupuncture points were selected individually according to the principles of Traditional Chinese Medicine (TCM), based on the patient's TCM diagnosis and the localization of headache pain. The individualized points included ST8 (Touwei), BL2 (Zanzhu), GB8 (Shuaigu), GB20 (Fengchi), BL12 (Fengmen), and EX-HN5 (Taiyang).

Sterile, single-use, disposable stainless-steel acupuncture needles (TeWa CB Type) of different diameters and lengths were used: 0.20 × 15 mm, 0.25 × 25 mm, and 0.25 × 30 mm. Needles were inserted to a depth generally ranging from approximately 5 to 35 mm, according to the anatomical characteristics and standard needling techniques of each acupuncture point. Superficial or oblique insertion was used for cranial and facial points, whereas deeper insertion was used for limb and muscular points. The insertion depth was adjusted according to the anatomical location of each point and the individual characteristics of the patient.

Manual stimulation was performed using a combination of rotation and lifting-and-thrusting techniques to elicit the characteristic de qi sensation. The achievement of de qi was assessed based on the patient's subjective report of characteristic sensations, including soreness, heaviness, and distension, during needle insertion and stimulation.

Needles were retained for approximately 30 min during each treatment session. Participants received a total of 10 acupuncture sessions, administered once weekly over a period of 10 weeks. All sessions were administered in an outpatient hospital clinic by trained and qualified medical acupuncturists, each with at least 10 years of clinical experience, in collaboration with the Bologna Association of Medical Acupuncturists (Associazione Medici Agopuntori Bolognesi-A.M.A.B.).

### Endpoints and assessments

The primary endpoint of the study was to evaluate plasma CGRP levels before and after a 10-session manual acupuncture cycle.

Secondary endpoint was exploring whether the time elapsed between the last migraine attack and blood sample collection influenced CGRP concentrations.

### Clinical variables

At baseline, demographic and clinical data were collected, including age, sex, migraine history, previous and concomitant migraine treatments, monthly migraine days (MMDs), and monthly acute medication intake.

Patients were instructed to fill in a paper headache diary throughout the study period to document migraine days and severity, with pain intensity recorded on a numerical rating scale from 0 to 10.

Participants also completed a validated instrument to assess migraine-related disability: the Headache Impact Test (HIT-6) (19). The questionnaire was completed at baseline and again at the follow-up visit, scheduled one week after the completion of the acupuncture treatment.

### Plasma collection and CGRP analysis

A venous blood sample was collected from each participant at baseline to measure CGRP levels. A second sample was obtained during the follow-up visit, scheduled one week after the completion of the acupuncture treatment cycle. All samples were collected during the interictal period, at the same time of day, between 8:00 and 10:00 am. None of the participants had taken acute triptans within the 48 h preceding either blood sampling. EDTA plasma samples were collected from the antecubital vein (3 mL), aliquoted, and stored at – 80 °C according to standard procedures.

CGRP plasma concentrations were measured using enzyme-linked immunosorbent assay kits (CGRP (human) ELISA kit #A05481 produced by Bertin Bioreagent, Montigny-le-Bretonneux, France). The assay was performed according to the specification of the manufacturer (20).

### Statistical analysis

Quantitative variables were expressed by mean ± standard deviation (SD) or median with interquartile range (IQR); categorical variables were presented with absolute number (*n*) and percentage (%).

Prior to comparative testing, the normality of the data distribution was examined using the Shapiro-Wilk test. A Wilcoxon signed-rank test, a non-parametric test suitable for within-subject comparisons, was used to determine whether there were significant changes in CGRP plasma levels, monthly headache days, analgesic consumption, and the HIT-6 scores from baseline and after acupuncture treatment cycle. Changes from baseline to follow-up time-points were considered statistically significant when *p*-value < 0.05.

Finally, in order to explore a possible association between CGRP plasma concentration and the time elapsed since the most recent migraine attack at the time of blood sampling, we performed a correlation analysis using Spearman's rank correlation coefficient (ρ). Data were analyzed using SPSS version 24 (IBM Corp., Armonk, NY, USA).

### Standard protocol approvals and patient consents

The study was conducted in accordance with the principles of good clinical practice and the study protocol was approved by the Local Ethics Committee of the local health service of Bologna, Italy (n. 19138/CE). All patients gave their written informed consent to study participation. Written informed consent was obtained from all enrolled patients, both for study participation and data publication. All procedures were conducted according to the latest version of the Declaration of Helsinki.

## Results

### Sample description

Seventy-one patients with episodic migraine were deemed eligible for inclusion. Nineteen were excluded due to protocol violations (details are provided in Figure 1). Analyses were therefore conducted on 52 patients (43 female and 9 men), with a median age of 45 years (IQR = 35.5–53). The diagnosis was migraine without aura in 46 patients (88.5%), while 6 patients (11.5%) had both migraine with and without aura. At the time of the study, 13 participants (25%) were receiving stable concomitant pharmacological prophylactic treatment. At baseline, the median migraine frequency was 8 (IQR = 7; 10) days per month, the median headache intensity was 7 (IQR = 6; 7) and the median use of acute medication was 10 (IQR = 7; 12) per month. Median HIT-6 score was 65 (IQR = 62; 69.5). Clinical characteristics are summarized in Table 1.

**Figure 1:**
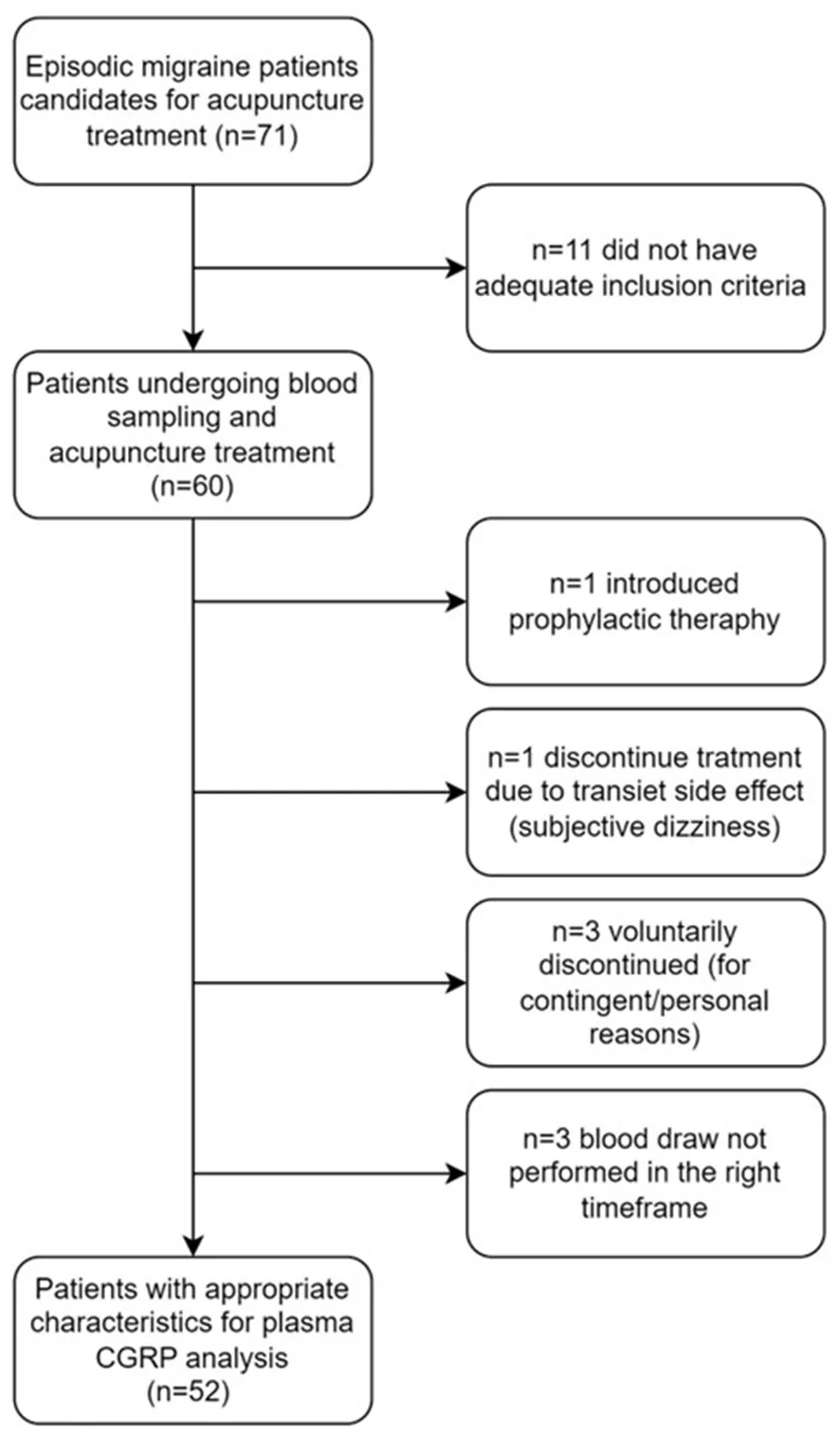
Study flow chart.

**Table 1:** Baseline characteristics.

Characteristic	Metric	Total
Sample	*N (%)*	52
Age, *y*	*Median (*IQR*)*	45 (35-53)
**Sex**		
Males	*N (%)*	9 (17.3)
Females	*N (%)*	43 (82.7)
**Migraine type**		
Migraine without aura	*N (%)*	46 (88.5)
Migraine with and without aura	*N (%)*	6 (11.5)
**Ongoing preventive treatment**		
Yes	*N (%)*	13 (25.0)
No	*N (%)*	39 (75.0)
**Type of ongoing preventive treatment**		
Tricyclic antidepressants	*N (%)*	6 (11.5)
Beta-blockers	*N (%)*	4 (7.7)
Calcium channel blockers	*N (%)*	2 (3.8)
Nutraceuticals	*N (%)*	1 (1.9)
**Migraine frequency (days/month)**	*Median* (IQR)	8 (7, 10)
**Migraine intensity (0-10)**	*Median (*IQR*)*	7 (6, 7)
**Acute medication intake (N/month)**	*Median (*IQR*)*	10 (7, 12)
**HIT-6 score**	*Median (*IQR*)*	65 (62; 69)
**Clinically relevant comorbidities**	*N (%)*	28 (53.8)
Endocrinological	*N (%)*	11 (21.2)
Psychiatric	*N (%)*	9 (17.3)
Vascular	*N (%)*	7 (13.5)
Autoimmune	*N (%)*	5 (9.6)
Gastroenterological	*N (%)*	3 (5.8)
**Ongoing pharmacological treatment for comorbidities**	*N (%)*	24 (46.2)

*N*, Number; IQR, interquartile range.

### Primary endpoint

Median CGRP levels were 60.9 (IQR = 55.6; 101.9) pg/ml at baseline, and 62.4 (IQR= 56.5; 90.6) pg/ml at the end of the study. A Wilcoxon signed-rank test showed no significant difference in CGRP levels between time points (*p* = 0.841). The median variation of the individual percentages change in CGRP levels from baseline was 0.28% (IQR = −8.2%; 6.8%). Table 2 and Figure 2 illustrate the change in plasma CGRP.

**Table 2:** CGRP plasmatic levels.

CGRP Plasma levels	Median (IQR)	Maximum value	Minimum value
Baseline (pg/ml)	60.9 (55.6–101.9)	667.9	15.1
Post-treatment (pg/ml)	62.4 (56.5–90.6)	610.8	19.0

IQR, interquartile range.

**Figure 2:**
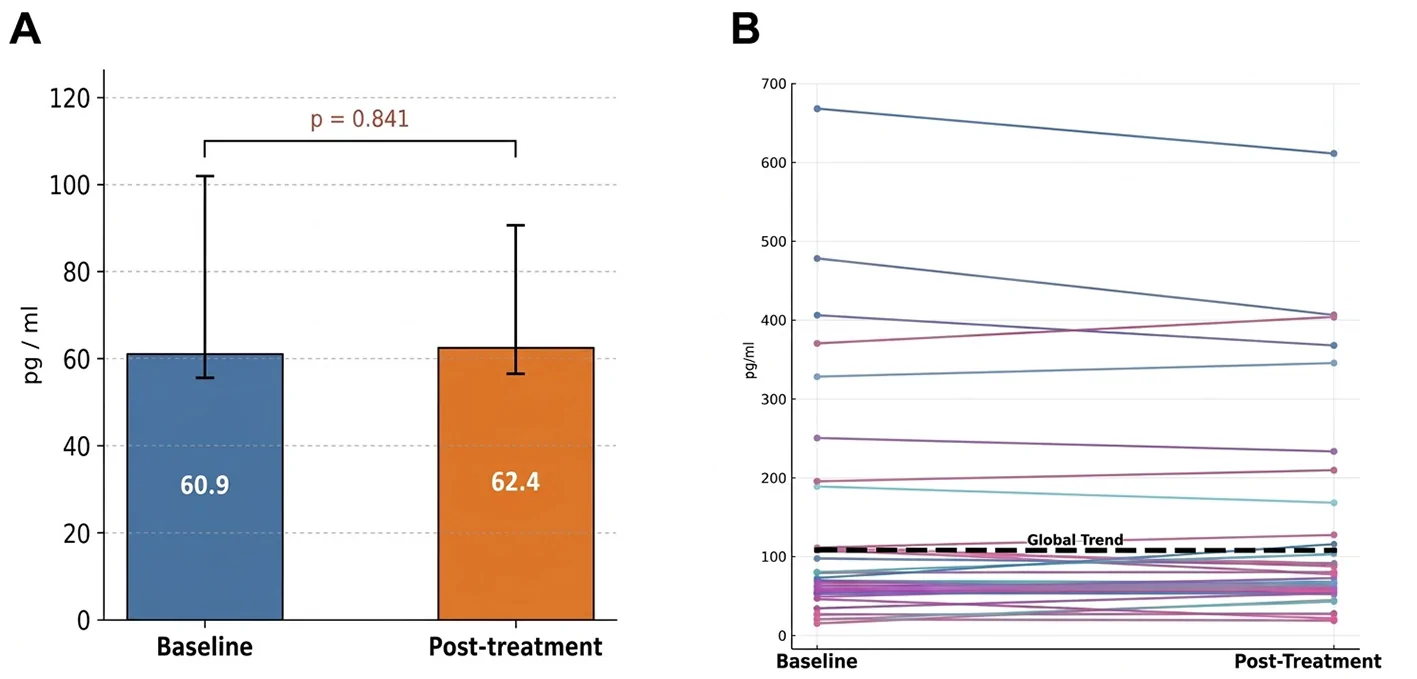
CGRP levels at baseline and post-treatment **(A)** Median CGRP levels at baseline and post-treatment, presented as bar plots with error bars indicating interquartile ranges (IQR). **(B)** Individual trajectories of CGRP levels from baseline to post-treatment. The dashed black line represents the global trend, defined as the overall change in CGRP levels from baseline to post-treatment across all participants, irrespective of individual trajectories.

### Secondary endpoint

No significant association was identified between CGRP concentrations, and the time elapsed since the most recent migraine attack at either baseline (ρ = −0.086, *p* = 0.549) or follow-up (ρ = 0.152, *p* = 0.282).

### Clinical outcomes of acupuncture treatment

After the completion of the acupuncture treatment cycle, the median migraine frequency was 7 (IQR= 6; 9) days per month, the median headache intensity was 6.2 (IQR = 4.5; 7) and the median use of acute medication was 7 (IQR = 4.3; 11) days per month. A Wilcoxon signed-rank test confirmed a statistically significant reduction in headache frequency (*p* < 0.001), migraine intensity (*p* < 0.001) and acute medication use (*p* < 0.001) following the acupuncture treatment cycle, compared to baseline. The median headache frequency decreased by 15.5% (IQR = −29.3%; 0%) between the two time points. Similarly, the median number of analgesics used per month decreased by 2 units (IQR = 0; 5), representing a 16.7% reduction, from a median of 10 at baseline (IQR = 7; 12) to 7 (IQR = 4.3; 11) after completing treatment.

Additionally, significant improvements were observed in patient-reported outcome measures. HIT-6 scores decreased from a median of 65 (IQR = 62; 69.5) at baseline to 60 (IQR = 55; 63) post-treatment, with a Wilcoxon signed-rank test confirming statistical significance (*p* < 0.001). This corresponds to a median change of−6 (IQR = −9; −1.5) points, representing a relative reduction of 8.7% (IQR = −13.1%; −2.3%).

Mild transient side effects (needle stick pain) were reported by 5 individuals (10%), but no serious adverse events occurred.

Clinical outcomes are summarized in Table 3 and Figure 3.

**Figure 3:**
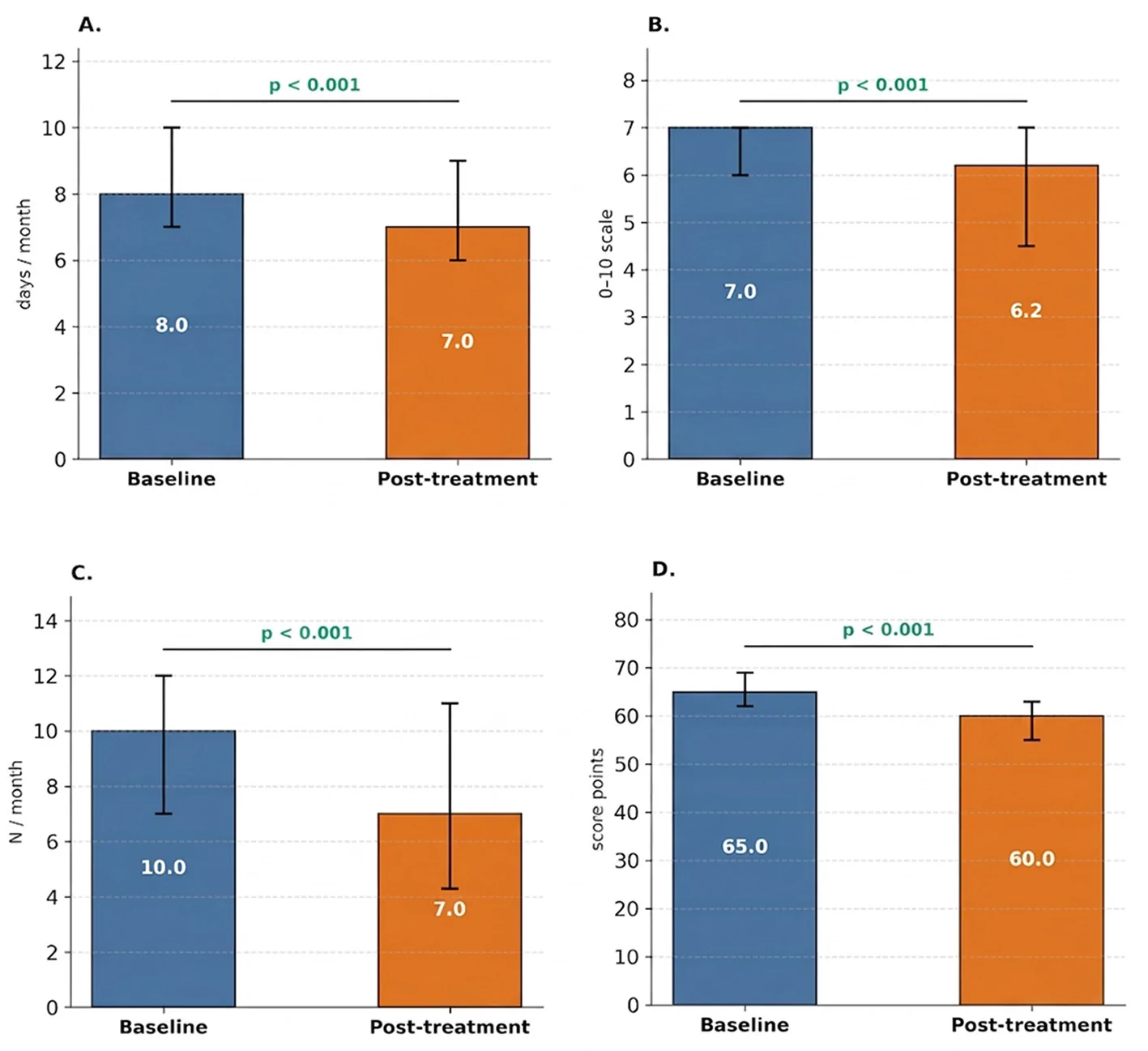
Changes in clinical outcomes between baseline and post-treatment. Bars represent median values with error bars showing interquartile ranges (IQR, 25^th^-75^th^ percentiles). **(A)** Migraine frequency (days/month). **(B)** Migraine intensity (0–10 scale). **(C)** Acute medication intake (doses/month). **(D)** HIT-6 score.

**Table 3:** Clinical Outcomes.

Outcome	Metric	Baseline	Post-treatment	p value
Migraine frequency (days/month)	*Median* (IQR)	8 (7, 10)	7 (6, 9)	< 0.001
Migraine intensity (0–10)	*Median* (IQR)	7 (6, 7)	6.2 (4.5; 7)	< 0.001
Acute medication intake (N/month)	*Median* (IQR)	10 (7, 12)	7 (4.3; 11)	< 0.001
HIT-6 score	*Median* (IQR)	65 (62; 69)	60 (55; 63)	< 0.001
CGRP levels (pg/ml)	*Median* (IQR)	60.9 (55.6–101.9)	62.4 (56.5–90.6)	=0.841

*N*, Number; IQR, interquartile range.

## Discussion

This study investigated longitudinal changes in plasma CGRP concentrations in patients with episodic migraine undergoing a 10-session course of manual acupuncture at a tertiary headache center.

Our results showed no significant changes in plasma CGRP levels between baseline and follow-up after the acupuncture treatment cycle (from 60.9 to 62.4, *p* = 0.841). The median individual percentage change in CGRP levels was 0.28% (IQR = −8.2%; 6.8%). These findings suggest that the clinical changes observed during the study period were not accompanied by a measurable change in peripheral plasma CGRP concentrations. The observed values of plasma CGRP were in line with previously reported values, although substantial significant interindividual variability has been described (11, 13, 21). Our findings are broadly consistent with the recent randomized, sham-controlled trial, which reported no significant between-group difference in changes in serum CGRP concentrations following acupuncture in patients with migraine without aura (16). However, direct comparison between the two studies should be made cautiously, as Sun et al. assessed serum rather than plasma CGRP, and methodological details regarding blood sampling and CGRP measurement were not reported. Indeed, several factors may contribute to the absence of a significant change in plasma CGRP. First, CGRP exhibits biological variability. CGRP levels are known to fluctuate significantly and can be influenced by temporal factors such as migraine phase, medication use, and time since the last attack (21–27). In this study, blood samples were collected 2.5 ± 2.0 days after the last attack at baseline and 3.5 ± 4.0 days at follow-up, which may have introduced variability in the measurements. Second, methodological limitations must be considered. A major concern is the substantial heterogeneity among commercially available assay kits, which differ in reference values and calibration curve methodologies used to quantify plasma CGRP levels (21, 26). This variability is compounded by the absence of standardized guidelines for CGRP measurement, making comparisons across studies challenging. Furthermore, peripheral plasma CGRP concentrations may not reliably reflect central neurochemical changes, where acupuncture is more likely to exert its therapeutic effects. Additionally, the substantial interindividual variability in CGRP levels may have further limited the ability to detect subtle treatment-related changes.

From a clinical point of view, we observed significant clinical improvements (*p* < 0.001) over the study period in terms of attack frequency, pain intensity, use of acute medications, and migraine-related disability. Although these improvements may be attributable, at least in part, to acupuncture, the single-arm design of the study prevents us from establishing a causal relationship, as placebo or nonspecific effects, regression to the mean, and natural fluctuations in migraine symptoms cannot be excluded. Additionally, the effects of acupuncture in migraine may be mediated through alternative mechanisms: modulation of pain processing pathways, autonomic nervous system regulation, and/or neuroimmune interactions (28, 29). In this regard, modulation of serotonergic pathways may represent a potential mechanism, as changes in serotonin levels following acupuncture have been reported both in migraine as well as in other pain conditions (30, 31).

This study presents several strengths and limitations that should be acknowledged. Among its strengths, the use of stringent inclusion criteria allowed for the recruitment of a relatively homogeneous patient sample, thereby increasing the internal validity and reliability of the clinical findings. Second, the study combined clinical assessments with a biological marker, allowing changes in clinical outcomes and plasma CGRP concentrations to be evaluated within the same cohort. Third, the acupuncture intervention was reported according to the STRICTA recommendations, with a standardized core prescription and individualized additional points, and all treatments were administered by experienced medical acupuncturists.

However, several methodological limitations should be acknowledged when interpreting the results. First, the absence of a control group represents a major limitation of the study design. As this was a single-arm study, the observed improvements in migraine frequency, pain intensity, acute medication use, and migraine-related disability cannot be attributed specifically to acupuncture. In particular, placebo and nonspecific effects, regression to the mean, and the natural fluctuation of migraine symptoms may have contributed to the observed clinical changes. Therefore, the present findings should be considered exploratory and hypothesis-generating rather than evidence of the clinical effectiveness of acupuncture. Controlled trials, ideally including a sham-acupuncture comparator and standardized methods for blood sampling and CGRP measurement, are needed to determine whether the observed clinical improvements are specifically attributable to the acupuncture intervention. Second, the relatively small sample size may have limited the statistical power to detect significant changes in CGRP levels or other subtle physiological effects. The stringent inclusion criteria, in the context of a tertiary headache center, resulted in slow recruitment, which limited the number of participants that could be enrolled. Larger studies are needed to confirm these findings and to investigate whether specific subgroups of patients may respond differently. Future studies could also assess CGRP in plasma alongside other biological fluids, such as saliva or tears, and explore the potential role of electroacupuncture. Finally, there was considerable variability in the timing of blood sample collection in relation to the last migraine attack. Since CGRP levels are known to fluctuate depending on the migraine phase, differences in sampling intervals (2.5 ± 2.0 days at baseline and 3.5 ± 4.0 days at follow-up) could have introduced additional variability, potentially obscuring actual changes in CGRP concentration over time.

## Conclusion

The role of peripheral plasma CGRP as a biomarker in migraine remains incompletely characterized, owing to difficulties in interpreting results related to sampling procedures, the lack of standardization among available assay kits, substantial interindividual variability in plasma CGRP levels, and the limited number of longitudinal studies. This study found no significant change in plasma CGRP levels between baseline and follow-up in patients with episodic migraine undergoing a 10-session acupuncture cycle. Although significant clinical improvements were observed across several clinical outcomes, the single-arm design does not allow these changes to be specifically attributed to acupuncture. The absence of a significant change in peripheral plasma CGRP suggests that it may not be a straightforward biomarker of the clinical changes observed in this setting. Further controlled studies with larger sample sizes and standardized CGRP sampling protocols are warranted to determine the specific clinical effects of acupuncture and to clarify the potential role of peripheral plasma CGRP as a biomarker of treatment response.

## Data Availability

The original contributions presented in the study are included in the article/supplementary material, further inquiries can be directed to the corresponding author.
